# A multi-state model to estimate incidence of heroin use

**DOI:** 10.1186/1471-2288-13-4

**Published:** 2013-01-14

**Authors:** Albert Sánchez-Niubò, Odd O Aalen, Antònia Domingo-Salvany, Ellen J Amundsen, Josep Fortiana, Kjetil Røysland

**Affiliations:** 1 Drug Abuse Epidemiology Research Group, IMIM- Institut de Recerca Hospital del Mar, Doctor Aiguader 88, E-08003, Barcelona, Spain; 2 CIBER in Epidemiology and Public Health, CIBERESP, Doctor Aiguader 88, E-08003, Barcelona, Spain; 3 Department of Biostatistics, Institute of Basic Medical Sciences, University of Oslo, P.O.Box 1122 Blindern, N-0317, Oslo, Norway; 4 Norwegian Institute for Alcohol and Drug Research, SIRUS, P.O. box 565 Sentrum, NO-0105, Oslo, Norway; 5 Department of Probability, Logic and Statistics, University of Barcelona, Gran Via de les Corts Catalanes 585, E-08007, Barcelona, Spain

**Keywords:** Back-calculation, Epidemiology, Heroin, Incidence, Multi-state model

## Abstract

**Background:**

Existing incidence estimates of heroin use are usually based on one information source. This study aims to incorporate more sources to estimate heroin use incidence trends in Spain between 1971 and 2005.

**Methods:**

A multi-state model was constructed, whereby the initial state “heroin consumer” is followed by transition to either “admitted to first treatment” or to “left heroin use” (i.e. permanent cessation or death). Heroin use incidence and probabilities of entering first treatment ever were estimated following a back-calculation approach.

**Results:**

The highest heroin use incidence rates in Spain, around 1.5 per 1,000 inhabitants aged 10–44, occurred between 1985 and 1990; subdividing by route of administration reveals higher incidences of injection between 1980 and 1985 (a mean of 0.62 per 1.000) and a peak for non-injectors in 1990 (0.867 per 1,000).

**Conclusions:**

A simple conceptual model for heroin users’ trajectories related to treatment admission, provided a broader view of the historical trend of heroin use incidence in Spain.

## Background

Recently, there has been increasing interest in ascertaining illegal drug use incidence for planning and evaluating prevention strategies [1]. In the case of heroin use, as survey data is not effective [2,3], incidence has been estimated from users eventually showing up mostly in treatment registers [1,4-9]. This incidence can be referred to as “problematic use incidence” and its trend could provide a satisfactory overview of problematic heroin use, assuming a constant proportion over total incidence.

The present study is an attempt to take the definition of problematic use incidence further by incorporating a proportion of individuals that used heroin who never show up in the main source under study (usually treatment). This proportion is based on other kinds of information, aggregated sources, estimates, or assumptions, such as mortality and cessation rates.

The idea is to study the unobserved entry (or immigration) of people to the state of “consumer”, based on a later first entry to treatment. Since heroin users may exit the state of consuming heroin before entering treatment, whether due to death or permanent cessation of their consumption, a “left heroin use” state is added to the model. The situation is thus represented by a set of mutually related states, in a so-called multi-state model with immigration.

This approach has parallels in studies in the HIV field, where a multi-state model was presented describing progression of HIV disease from infection to AIDS in several stages [10,11] or Rossi’s dynamic “mover-stayer” model as a theoretical approach applied to simulate a complete drug user “career” [12,13].

We shall use a back-calculation type approach to estimating the incidence. This is similar in spirit to de Angelis et al. [14]. However, following the presentation of Aalen et al. [10], we have found it useful to display the approach as a simple multi-state model.

To our knowledge, previous approaches had to assume that treatment availability was stable over time [1,4-9]. As treatment data is usually only available for a limited period of time, a lag time distribution between heroin use onset and first treatment ever has been employed to avoid underestimating incidences. However, this assumption may be too strong if important changes in treatment availability had occurred. Therefore, the back-calculation approach used in this study avoids this assumption.

In Spain, heroin use generated important health problems in the eighties and early nineties [15], thereafter all indicators (mortality, treatment admissions, hospital emergencies, surveys, etc.) showed a decreasing trend until 2006 [16]. Efforts to calculate incidence of heroin use have been and still are considerable to understand the overall trend and assess its consequences [1]. To date only one previous study has estimated heroin use incidence in Spain. However, due to important changes of treatment availability, the reliability of the estimation was questionable [6].

The multi-state model approach may be successfully used to estimate heroin use incidence with the available Spanish heroin users’ treatment data. We use the same treatment database as the previous study to assess differences in the incidence estimates depending on the approach used.

The objective of the present study was to estimate heroin use incidence in Spain through a multi-state model with immigration and assess differences with previous study’s estimates.

## Methods

### Incidence estimation: the multi-state model with immigration

**Figure 1:**
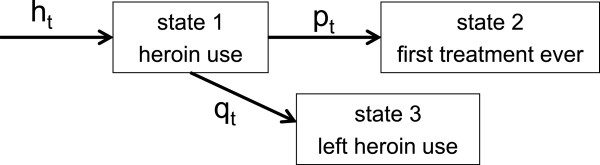
**Multi-state model diagram.** Parameter *h*_*t*_ denotes immigration of individuals starting heroin use, *p*_*t*_ transition rate entering treatment for the first time, and *q*_*t*_ transition rate leaving heroin use without entering treatment, at time *t*.

We will describe a heroin user’s trajectory simply using the time of transition from state 1 (first heroin use) to one of two possible subsequent states: first treatment ever (state 2), or leaving heroin use before any treatment, either by permanent cessation or death (state 3) (Figure 1).

We shall now formulate our back-calculation type model. We let *t* be the calendar time and assume that the number of new heroin users per unit of time is Poisson distributed. The Poisson assumption is standard and well justified on statistical grounds [14]. Moreover, we let *h*_*t*_ denote the expected number of people entering state 1 (heroin use) at *t*. The number *p*_*t*_ denotes the probability that a given heroin user initiates their first treatment ever at time *t*, given that they were in state 1 at the previous time. We assume this probability independent of the era when their heroin use began. The number *q*_*t*_ denotes the probability of an individual leaving heroin use at time *t*, given that they were in state 1 at the previous time. The cause could be death or other permanent cessation of heroin use. We want to estimate the parameters *h*_*t*_, the expected number of new heroin users at time *t*. The probability *q*_*t*_ is assumed known.

We now construct the likelihood function. This is a standard Poisson type likelihood, following the approaches in the basic back-calculation papers [14]. However, since our model is here adapted to the particular data we have, we briefly present the necessary formulas. Let *N*_*ij*_ be the observed number of individuals that start heroin use in year *i* and enter first treatment in year *j*. Let *μ*_*ij*_ be the expected value of *N*_*ij .*_ A short computation shows that:

(1) $$\mu_{\mathrm{ij}}=h_{i}\cdot \prod_{k=i}^{j-1}\left(1-p_{k}-q_{k}\right)\cdot p_{j}$$

We see that *μ*_*ij*_ is the product of the expected number of new heroin users in year *i* (*h*_*i*_), the probability that a given heroin user remains in state 1 from time *i* to time *j-1* and the probability of a transition from state 1 to state 2 at time *j*. It also follows that each *N*_*ij*_ is Poisson distributed. This gives us the following simple expression for the likelihood:

(2) $$L={\prod_{i,j}\mu_{\mathrm{ij}}}^{N_{\mathrm{ij}}}\cdot \exp\left(-\mu_{\mathrm{ij}}\right)$$

Maximizing this likelihood yields estimates for *h*_*t*_ and *p*_*t.*_

### Treatment data

The Spanish Drug Observatory maintains a drug information system. Its indicator “treatment” is based on data from all treatment starts in public and publicly funded centres. The health coverage is universal and all kinds of treatment are considered. In the present study, we included 169,257 persons who entered first treatment for heroin use from 1991 to 2006 when aged 15 to 54 (mean 28 years), and who started their heroin use between 1971 and 2006 when aged 10 to 44 (mean 21 years). The database was split into two subsets: people who declared injection as the most frequent route for heroin use in the last 30 days before first treatment admission (29%) and people who declared using routes other than injection (68%). Route of administration was missing in 3% of persons.

### Assumptions about parameters

As in all applications of back-calculation where a detailed history of individuals is not observed [14], we have to make some simplifying assumptions, presented below.

#### First treatment data for the period 1971–1990

In the estimation of *p*_*t*_, first treatment data was available for *t* between 1991 and 2006, thus restricting estimates to this period. For the preceding part of the study period (*t* in 1971–1990), we made an educated guess of *p*_*t*_ based on general heroin use information in Spain. Based on the first appearances of admissions for heroin use in the emergency units in Spain in 1982 [17-19]; we assumed probabilities of entering treatment (*p*_*t*_) as low as 0.01 between 1971 and 1981, as there were still no specific treatments available. Thereafter we assumed a linear increase to the value estimated for the parameter *p*_*t*_ in 1991.

#### Mortality for heroin users

Mortality rates for heroin users were only available from two local cohort studies covering the period 1985 to 1999 [20,21] in an area where injecting was the predominant route of administration [22]. As we did not have better approximation, yearly rates from these studies (minimum 1.4% in 1985, maximum 6.6% in 1995) were extrapolated to the whole country for the corresponding year. For the period 1971 to 1984 a smooth increasing trend from a mortality rate of 1% to 1.4% was applied. Mortality rates from 2000 to 2006 decreased from a rate of 1.5% in 1999, to 1% in 2006. In the analysis by route of administration the same mortality rate was used for injectors, but a constant mortality rate of 1% for non-injectors since they have lower risk [23].

Degenhardt et al. reported a pooled crude mortality rate of 2.09 per 100 person-years and that mortality risk was increased among out-of-treatment heroin users [24]. In a sensitivity analysis (see analysis section) we considered alternative mortality rates obtained by adding 0.01 to the yearly mortality rates in order to ensure a minimum rate of at least 2%. Note that, in the multi-state model we are imputing mortality rates before first treatment.

#### Cessation rates

Owing to the impossibility of obtaining permanent cessation rates, we looked for lasting cessation rates from long-term cohort studies. As such studies are not available in Spain, we considered yearly cessation rates from a thorough review which reported a range of 0.02-0.04 [25]. Our analyses considered these two extreme values.

### Analysis

For all heroin users and for injectors and non-injectors separately, we applied the aforementioned multi-state model with the Spanish treatment data and the assumed leaving rates to estimate the heroin use incidence (*h*_*t*_, *t* ranging from 1971 to 2006). As explained, the model also estimates the probability of entering first treatment (*p*_*t*_, *t* in range 1991 to 2006). We considered the yearly cessation rate of 0.04 and the non-modified mortality rate derived from the local cohort studies. Note that the probability of leaving drug use without having ever been registered for first treatment (*q*_*t*_) is the sum of the cessation and mortality rates for each year from 1971 to 2006.

In equation 1 when *i=j,* it means that users began treatment in the same year as they started heroin use. This gives on the average about half a year of observation, and so we must weight *μ*_*ij*_ by 0.5.

To assess the fit of the expected incidence values *μ*_*ij*_ with their observed values N_ij_, we have drawn their curves stratified by year of heroin use onset (*i*).

As results can be dependent on assumptions, a sensitivity analysis was performed to evaluate the two chosen mortality and cessation rates obtaining four combinations of *q*_*t*_, that are reflected in four curves of estimated incidence rates. These combinations were: firstly and as a matter of choice, the available mortality rates and a yearly cessation rate of 0.04; secondly, the same mortality rates and a yearly cessation rate of 0.02; thirdly, the same mortality rates modified by adding 0.01 to the rate for each year and a yearly cessation rate of 0.04; and finally, the modified mortality rates and a yearly cessation rate of 0.02.

Statistical uncertainty was estimated using a bootstrap technique with 500 re-samples, where each re-sample was made up of two parts: 1) the treatment database was re-sampled with replacement and, 2) both our “best guesses” for *p*_*t*_ in the period from 1971 to 1990, and the cessation rate for all years were sampled from gamma distributions. The shape and scale parameters were derived from the mean and standard deviation, taking the mean as the “best-guess” value, and the standard deviation was established as 0.01.

The expected number of new heroin users per year was obtained, and converted into rates per 1,000 inhabitants, based on Spanish population yearly census data for people aged 10-44 [26].

Incidence estimates from the previous study were retrieved to compare with the present estimates. Both the period of years covered and the census figures were the same.

The software used for the statistical analysis was R version 2.13.0 [27]. The study was approved by the Ethics Committee of the Institution with number 2004/1828/I.

## Results

### Estimates of incidence and probability of entering treatment

**Figure 2:**
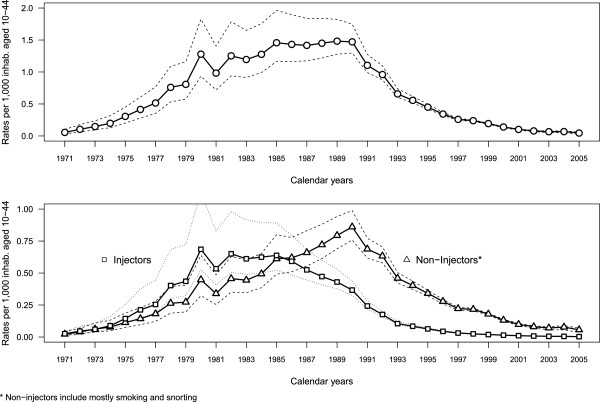
Estimated heroin use incidence rates*: global (upper graph) and by route of administration** (lower graph). * rates per 1,000 inhabitants aged 10–44, in Spain, with 95% confidence interval, ** the most frequent route of administration in the last 30 days before entering treatment.

Applying the multi-state model with immigration yielded estimated probabilities of entering treatment for the first time (*p*_*t*_) which exhibited an overall increasing trend from 0.08 (95% CI 0.07-0.09) in 1991 to 0.29 (95% CI 0.23-0.49) in 2005. Incidence estimates of general heroin use and by route of administration with 95% confidence intervals are plotted in Figure 2. For general heroin use, the highest incidences were between 1985 and 1990 with rates around 1.5 new heroin users per 1,000 inhabitants aged 10–44, followed by a steep decline from 1991 to 1997, then a more gradual decrease from 1998 (0.24 per 1,000) to 2005 (0.05 per 1,000).

**Figure 3:**
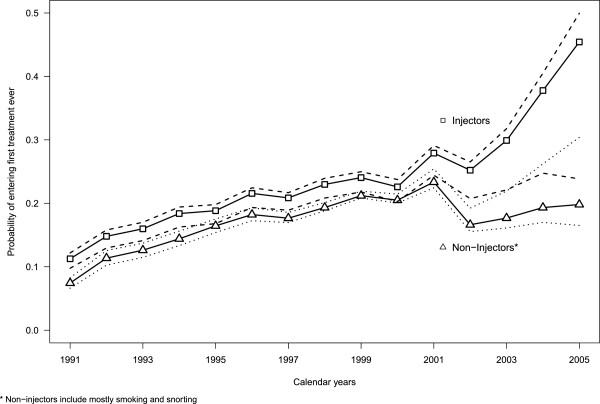
**Estimated probabilities of entering first treatment ever (*p***_***t***_) in Spain by route of administration**, with 95% confidence interval. ** The most frequent route of administration in the last 30 days before entering treatment.

In the analysis by route of administration the probability of entering first treatment for heroin users declaring injection was higher than for non-injectors between 1991 and 2000 with a difference of around 0.03. However, after 2001 this difference increased progressively (Figure 3). Incidence rates for injectors were higher than for non-injectors until 1985 and lower thereafter (lower graph in Figure 2). For injectors the highest values were observed between 1980 and 1985 (a mean of 0.62 per 1,000) whereas for non-injectors the peak was in 1990 (0.86 per 1,000).

**Figure 4:**
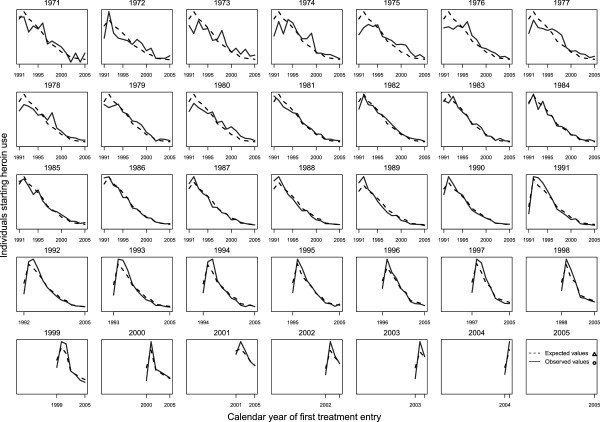
Observed and expected number of individuals by heroin onset cohort from 1971 to 2005, and entering first treatment ever from 1991 to 2005, in Spain.

Comparing the curves of the expected incidence values *μ*_*ij*_ with their observed values N_ij_, by year of heroin use onset (*i*), we assessed that the fit was good (Figure 4).

**Figure 5:**
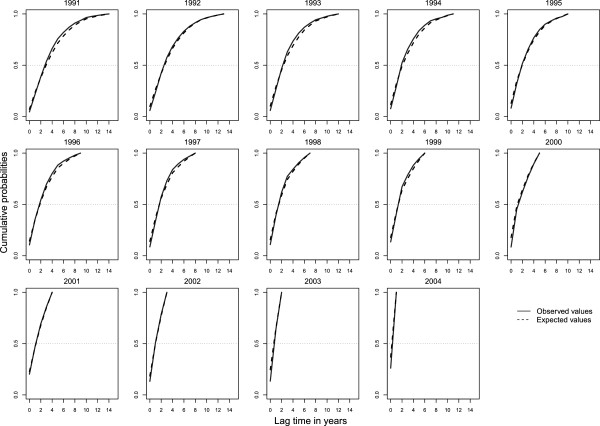
**Observed and expected distributions of lag time between heroin use onset and first treatment ever conditional on treatment starting before 2006.** Distributions are given by heroin onset cohort from 1991 to 2004, in Spain.

We could also check this good fit modifying the observed values as *N*_*i,j-i*_ and the expected values as *μ*_*i,j-i*_ , where *j-i* represents the lag time between drug use onset *i* from 1991 to 2004 and first treatment ever *j*, conditional on treatment starting before 2006 (Figure 5).

### Sensitivity analysis

**Figure 6:**
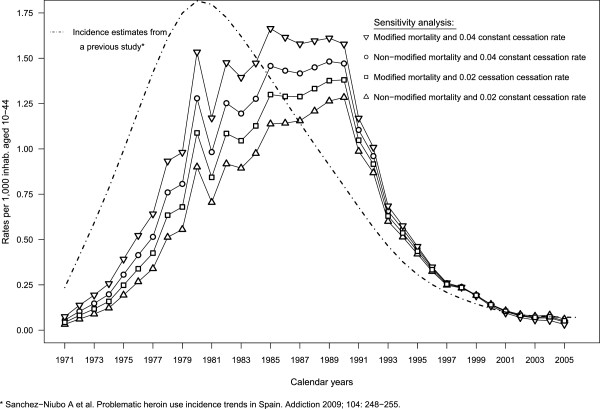
Sensitivity analysis* of heroin use incidence rates**, and estimated incidence rates from a previous study***. * combining two cessation rates and two mortality rates, ** per 1,000 inhabitants aged 10–44, in Spain, *** Sánchez-Niubò A et al. Problematic heroin use incidence trends in Spain. Addiction 2009; 104: 248–255.

Each combination of mortality and cessation rates produced large variations in the eighties when incidence was highest (Figure 6). Note that lower cessation and mortality rates yielded lower incidence rates, and vice versa.

Regarding probabilities of entering treatment for the first time, from 1991 to 2000 estimates only varied slightly, with a maximum difference of only 0.02 (data not shown). From 2001 differences increased progressively reaching a final value of 0.2 in 2005 between the lowest and highest combinations of cessation and mortality rates (probability of 0.22 versus 0.42).

Confidence intervals for the various estimates overlap, except for the combination yielding the lowest estimates (results not shown).

### Comparison of incidence estimates

Incidence estimates from a previous study [6] are shown in Figure 6. These estimates had an earlier peak around 1980 and, although they were lower than present ones in the 90’s, they overtook them in the last few years.

## Discussion

We have established a conceptually simple multi-state model to obtain estimates for the incidence rates of heroin use and applied it over a long period in Spain. The highest incidences were observed from 1980 to 1985 corresponding to injectors and a peak in 1990 to non-injectors.

In comparison with previous studies, our estimates are wider in scope since by including mortality and other permanent cessation into the multi-state model it is possible to account for almost all problematic heroin users after drug use onset.

The conceptual model employed in this study focuses on the first phase of a heroin user’s “career”: from heroin use initiation to treatment. Other more complete models based on a theory of compartmental epidemic models over drug user “career” have also been described [12]. Adding more states into the model using the data available would, however, make estimating incidence too complex because heroin use cessation and relapse are frequent and difficult to follow up. Knowing first entry to treatment and first use, we only need to account for quitting heroin use before first treatment through complete recovery (cessation) or death.

As a Spanish anthropologist described, in Spain heroin use had its first phase in the years 1977 and 1978, when the first users became visible, being endemic in the second phase between 1979 and 1982, and reaching its zenith between 1983 and 1986 leading to the institutionalization of the problem [18]. Therefore, trends in overall heroin use incidence obtained in the present study seem reasonable and consistent with previous knowledge about the Spanish heroin epidemic and the HIV-AIDS epidemic [15]. Specifically, the inflection point observed in 1985, when the incidence rate of injected heroin use fell below the rate for non-injection, is consistent with the trend of decreasing HIV incidence among injectors in Spain. However, we observe that estimates from the previous study had higher incidence figures earlier than the present ones (Figure 6). They reflect the fact that the availability of treatment was assumed stable throughout the entire period, leading to high estimates too soon.

We found a decreasing trend in the incidence estimates for the last years observed, which is probably related to the decreasing trend observed in all indicators towards the end of the period studied, as mentioned in the introduction. However, estimates for these last years from the previous study became stable overtaking the estimates from the present one (Figure 6). This is due to the two studies employing different approaches. Equation 1 in the present study was formulated assuming that *p*_*t*_, the probability of entering first treatment, was independent of the era when a person's first heroin use began. Actually, this would be not entirely true if lag time between the drug use onset and first treatment followed a determined pattern, as previous studies assumed [1,6]. However, if we observe Figure 5, the lag time distribution for the observed values *N*_*i,j-i*_ and for the expected values *μ*_*i,j-i*_ (*j-i* represents the lag time), for each year of heroin use onset from 1991 to 2004 all fitted well. So, to modify the equation 1 including the probability of entering first treatment conditional on the initiation of heroin use would be too complex and may not have great practical importance. Therefore, bias can be inherent in both the independence assumption and assuming a determined pattern of lag time, although the direction of such bias cannot be determined.

As observed by de Angelis [14], results are dependent on assumptions. However, the sensitivity analysis showed that the different incidence curves generated by varying the cessation and mortality rates had similar shapes (i.e. trends) although different levels, suggesting that the model’s estimates were stable. Moreover, we observed that the confidence intervals of incidence figures estimated using non-modified mortality rates and a cessation rate of 0.04 completely contain those of the other three estimates. Thus the chosen incidence estimates do not differ significantly from the other three estimates, which resulted from varying the rates involved.

Concerning the assumptions made about the model parameters, such as 1) mortality rates, 2) permanent cessation, both before first treatment and 3) heroin users that started their first treatment before the observation period, we need to consider possible limitations:

1) Mortality rates were extrapolated by applying to the whole country figures from the North-East of Spain where heroin use injection was more frequent than in the rest of the country [22]. The extrapolation appears to be appropriate, since the period where the highest mortality rates are found for the two cohorts studied (1985 to 1999) coincides with the period when there were more HIV and drug injection related deaths in Spain [16]. However, if the extrapolation is not appropriate it would lead to over-estimation of the total incidence of heroin consumption for the whole country. Note that adding an additional 0.01 to the yearly mortality rates, i.e. to account for the risk of dying when out of treatment being greater, would lead to even greater over-estimations of the incidence.

2) Using lasting cessation rates from long-term cohort studies would overestimate incidence as they include persons with long cessation periods who finally may relapse. On the other hand, the fact that experimental users were not included in studies estimating cessation rates would produce underestimates. Nevertheless, these experimenters are only of anecdotic value for policy interventions.

3) Although the exact dates and figures we have taken for first treatment probabilities prior to the observation period may not apply to the whole country, the values assumed seem plausible as we obtained an increasing sequence of probabilities from 1982 up to the first one estimated based on observed data in 1991, which happens to have a similar slope to that estimated in the observed period.

Besides model building, it is important to consider other limitations related to treatment data, both to its overall availability and to its accuracy. Treatment register data covers public and publicly funded centres, missing people using private treatment centres. This entails a small proportion especially once public substitution treatment centres were widely implemented all over the country following legislation in 1990 [28]. In relation to treatment variables used, we acknowledge the possibility of error in the reported year of heroin use onset, in which we cannot discern any systematic trend except perhaps a certain propensity to round to years ending in 0 or 5.

Incidence trends by route of administration do not necessarily reflect the route used at the time of onset, as the variable was collected referring to the 30 days prior to first treatment. However, in a previous study involving heroin users, both in and out of treatment, and a mean length of use of 10 years, more than 50% did not change their initial route of heroin administration [29]. Thus the study of incidence trends by route of administration in the period immediately previous to first treatment can provide an idea of the different patterns of heroin administration during the heroin epidemic in Spain [30]. The higher probability of entering treatment among individuals declaring injection in the previous month may possibly be related to a change to a more harmful route of administration.

## Conclusions

With a simple conceptual model of heroin users’ trajectories related to treatment demand, it has been possible to obtain approximations of heroin use incidence trends. Moreover, different assumptions made do not systematically skew the conclusions. However, enhancing accuracy of drug users’ trajectories and an updating of new treatment admissions will further contribute to better incidence estimates.

## Abbreviations

(HIV): Human immunodeficiency virus; (AIDS): Acquired immunodeficiency syndrome; (CI): Confidence interval.

## Competing interests

The authors declare that they have no competing interest.

## Authors’ contribution

Albert Sánchez-Niubò participated in the study design, proceeded with data analysis and writing of the manuscript draft; Odd O. Aalen contributed to the design of the method and helped in the writing of the methodological part; Antònia Domingo-Salvany contributed to data acquisition, helped with the interpretation of the data and revised critically the draft for important intellectual content; Ellen J. Amundsen helped with the interpretation of the data and revised critically the manuscript for important intellectual content; Josep Fortiana helped in the analysis of the data and participated in the writing of the methodological part of the work; Kjetil Røysland designed the method implementation, supervised all the work and revised critically the manuscript. All authors approved the final version of the manuscript.

## Pre-publication history

The pre-publication history for this paper can be accessed here:

http://www.biomedcentral.com/1471-2288/13/4/prepub
